# Anemia is an independent prognostic factor in intracerebral hemorrhage: an observational cohort study

**DOI:** 10.1186/cc12827

**Published:** 2013-07-23

**Authors:** Joji B Kuramatsu, Stefan T Gerner, Hannes Lücking, Stephan P Kloska, Peter D Schellinger, Martin Köhrmann, Hagen B Huttner

**Affiliations:** 1 Department of Neurology, University of Erlangen-Nuremberg, Schwabachanlage 6, 91054 Erlangen, Germany; 2 Department of Neuroradiology, University of Erlangen-Nuremberg, Schwabachanlage 6, 91054 Erlangen, Germany

**Keywords:** Intracerebral haemorrhage, Spontaneous ICH, On admission anemia, Unfavorable outcome, Outcome predictor

## Abstract

**Introduction:**

To date only two studies have evaluated anemia status in acute intracerebral hemorrhage (ICH) reporting that on admission anemia (OAA) was associated with larger hematoma volume, and lower hemoglobin levels during hospital stay, which related to poorer outcome. The question remains whether anemia influences outcome through related volume-effects or itself has an independent impact?

**Methods:**

This single-center investigation included 435 consecutive patients with spontaneous ICH admitted to the Department of Neurology over five years. Functional short- and long-term outcome (3 months and 1 year) were analyzed for anemia status. Multivariate logistic and graphical regression analyses were calculated for associations of anemia and to determine independent effects on functional outcome. It was decided to perform a separate analysis for patients with ICH-volume <30cm^3 ^(minor-volume-ICH).

**Results:**

Overall short-term-outcome was worse in anemic patients (mRS[4-6] OAA = 93.3% vs. non-OAA = 61.2%, *P *< 0.01), and there was a further shift towards an increased long-term mortality (*P *= 0.02). The probability of unfavorable long-term-outcome (mRS[4-6]) in OAA was elevated 7-fold (OR:7.5; *P *< 0.01). Receiver operating characteristics curve (ROC) analysis revealed a positive but poor association of ICH-volume and anemia (AUC = 0.67) suggesting volume-undriven outcome-effects of anemia (AUC = 0.75). Multivariate regression analyses revealed that anemia, besides established parameters, has the strongest relation to unfavorable outcome (OR:3.0; *P *< 0.01). This is even more pronounced in minor-volume-ICH (OR:5.6; *P *< 0.01).

**Conclusions:**

Anemia seems to be a previously unrecognized significant predictor of unfavorable functional outcome with independent effects beyond its association with larger hemorrhage volumes. The recognition of anemia and its treatment may possibly influence outcome after ICH and as such prospective interventional studies are warranted.

## Introduction

Anemia is a common finding among intensive care patients, with 95% being anemic by day 3 and up to 50% receiving red blood cell (RBC) transfusions during their stay [1]. For neurocritical care patients, most research has been conducted for traumatic brain injury and subarachnoid hemorrhage, documenting associations for lower Hb-levels with poorer outcome, but large randomized controlled trials (RCT) are missing [2-4]. Despite existing evidence in general intensive care the appropriate treatment strategy for these brain-injured patients still needs to be elucidated [2,5].

For intracerebral hemorrhage (ICH) only three studies are available that analyzed anemia, though including heterogeneous patient populations, for example, ICH related to oral anticoagulants. Diedler and colleagues found an association of a nadir hemoglobin level during hospital stay with functional outcome in supratentorial ICH [6]. Kumar and co-workers identified on-admission anemia (OAA) to be independently associated with increased hemorrhage volumes and multivariate analysis suggested that anemia might have effects on outcome not related to ICH volume [7]. Another recent study investigated the influence of RBC transfusions during acute ICH treatment and reported improved survival rates at 30 days though transfusion regimes have not been investigated [8].

It remains unclear whether anemia is a marker for moribund patients with a poor prognosis, or if anemia directly leads to increased hemorrhage volumes impacting outcome [6,7]. Given this controversy, the present study sought to investigate the impact of anemia on functional outcome in solely spontaneous ICH patients. Based on the strong association of ICH volume and outcome, and in light of large hemostatic and blood pressure lowering trials failing to influence clinical endpoints after enrolling patients with ICH volumes up to 60 cm^3 ^[9,10], defining rather tighter volume cutoffs - as done in the ongoing CLEAR-IVH trial (ICH volume <30 cm^3 ^[11]) - appears reasonable. As anemia represents an easily treatable medical condition, which, if compensated, may possibly influence functional outcome, we performed a sub-analysis in 'minor-volume' ICH (that is, <30 cm^3^) to minimize volume-based outcome effects and to evaluate the relevance of anemia in this special cohort.

## Materials and methods

### Patient selection

The study was performed in accordance with ethical standards and was approved by the ethics committee of the Friedrich-Alexander University Erlangen-Nuremberg, Germany. Informed consent was obtained from all patients or legal representatives. All consecutive patients with spontaneous ICH admitted to the Department of Neurology over a 5-year period (2006 to 2010) were retrospectively analyzed from our prospective institutional database. By definition, we excluded secondary ICH; that is, ICH related to oral anticoagulant therapy, trauma, tumor, arteriovenous malformations, central venous thrombosis, subarachnoid hemorrhage, or ICH after acute thrombolysis or coagulopathy (platelet counts <50.000/μL; INR >1.4). Altogether, 464 spontaneous ICH patients were identified from our database, 29 patients were lost to follow-up or refused consent. Hence, 435 (93.7%) patients of central European descent remained for final analysis. We *a priori *decided to perform a separate analysis for those patients of our cohort who showed a parenchymal ICH volume <30 cm^3 ^(*n *= 267; in the following referred to as 'minor-volume' ICH patients).

### Parameter acquisition

In-hospital parameters (Glasgow Coma Scale (GCS), National Institute of Health Stroke Scale (NIHSS)), ICH score, blood pressure, mechanical ventilation, length of stay, external ventricular drainage (EVD) for occlusive hydrocephalus [12], diagnosis of pneumonia, urinary tract infection, and sepsis according to established criteria [13-15] were obtained by reviewing the patient's medical charts and institutional databases. Initial laboratory parameters were retrieved from our laboratory database. Anemia was defined according to WHO definitions (Hb <12 mg/dl for women, Hb <13 mg/dl for men) [5,16]. Further, OAA will be referred to as anemia within the text and for reasons of brevity as OAA in tables and figures.

Patient characteristics (history of ischemic or hemorrhagic stroke, cardiovascular events as myocardial infarction), use of nicotine, alcohol abuse, diagnosis of arterial hypertension, diabetes mellitus, and hypercholesterolemia - according to established criteria [17-19] - were evaluated by a standardized mailed questionnaire. A semi-quantitative phone interview with patients or their closest relatives was conducted whenever this questionnaire did not return within 4 weeks. In cases of insufficient data retrieval (*n *= 39 patients; no correlation with neurological outcome), primary care physicians were contacted by telephone interview. The 435 investigated patients responded in 73.8% (*n *= 321) by questionnaires and 26.2% (*n *= 114) by telephone interviews. Two physicians (JBK, SG) trained and certified for data collection on disability and quality of life performed these interviews.

### Outcome measures

Mortality and functional outcome 90 days (short-term outcome) and 1 year (long-term outcome) after disease onset were obtained as described above and evaluated using the modified Rankin scale (mRS) [20]. A favorable outcome was defined as a mRS between 0 and 3, an unfavorable outcome as a mRS of 4 to 6. Moreover, a 'shift' from short- to long-term outcome was analyzed separately for the entire cohort as well as for minor-volume ICH patients.

### Imaging

Diagnosis was made either by computed tomography (SIEMENS Somatom Volume Zoom, Germany) or magnetic resonance imaging (SIEMENS Sonata, 1.5 Tesla, Germany). Two neuroradiologists (HL and SK) blinded to clinical data reviewed the scans randomized and independently. For discrepancies a second consensus analysis was made. Hematoma growth (volume >33% [21]) was determined upon follow-up imaging which was performed within 24 ± 6 h. Parenchymal ICH volume was calculated by the formula of ellipsoids (ABC/2) [22] and imaging modalities were compared using a validated conversion model [23]. IVH was documented and scored by Graeb score summation (GS) [24], and midline shift was measured in mm.

### Statistical analysis

Statistical analysis was performed with SPSS 19.0 [25]. The significance level was set at α = 0.05. Statistical tests were two-sided. The Kolmogorov-Smirnov test was applied to determine distribution of the data. Latter are presented as mean ± SD (compared using the student T-test), or as median and interquartile range (IQR) (compared using the Mann-Whitney U-test), as appropriate. The Pearson-χ^2 ^and the Fisher's exact tests were used to compare frequency distributions of categorized variables between anemic and non-anemic patients. The correlation of anemia with hemorrhage volume and functional outcome was graphically analyzed by the receiver operating characteristics curve (ROC) [26]. The influence of ICH volume on functional outcome (OAA *versus *non-OAA) was analyzed by graphical regression analysis, the locally weighted scatterplot smoothing (LOWESS) with a = 0.25-0.5. A sub-analysis for minor-volume ICH (ICH volume <30 cm^3^) patients was undertaken to discriminate the effects of anemia from the overwhelming impact of ICH volume. For prediction of unfavorable long-term outcome (mRS = 4-6) two step-wise forward inclusion multivariate logistic regression models were calculated, one for the entire cohort and one for minor-volume ICH patients. For both models all investigated parameters were first tested univariately. Consecutively, parameters reaching a statistical trend in univariate analysis (that is, *P *< 0.1) were included into the multivariate models.

## Results

### Anemic patients have larger baseline volumes and poorer neurological status

All evaluated parameters dichotomized for OAA *versus *non-OAA are given in Table 1. Overall, 24.1% of patients were anemic on admission and as key findings showed significantly larger ICH volumes (48.6 cm^3 ^*versus *15.0 cm^3^; *P *< 0.01) and neurological status was considerably poorer (*P *< 0.01). There were no co-morbidities that revealed a significant association with anemia.

**Table 1:** Demographic-, baseline,-, neuroradiological-, laboratory, treatment characteristics for all ICH patients with OAA *versus *non-OAA.

Spontaneous ICH (*n *= 435)	OAA (*n *= 105)	Ø OAA (*n *= 330)	*P *value
Age^a ^(years)	71.4 (± 12.4)	69.1 (± 11.9)	0.09
Gender^b ^(women)	49 (46.7%)	147 (44.5%)	0.70
			
Prior medical history			
Pre-mRS^c^	1 (0-3)	1 (0-3)	0.14
Hypertension^b^	80 (76.2%)	261 (79.1%)	0.34
Diabetes^b^	25 (23.8%)	83 (25.2%)	0.45
Hypercholesterolemia^b^	26 (24.8%)	94 (28.5%)	0.27
Ischemic stroke^b^	28 (26.7%)	63 (19.1%)	0.10
Hemorrhagic stroke^b^	9 (8.6%)	33 (10.0%)	0.40
Cardiac event^b^	14 (13.3%)	52 (15.8%)	0.33
Alcohol abuse^b^	21 (19.6%)	60 (15.3%)	0.39
Smoking^b^	30 (28.6%)	111 (33.6%)	0.20
Antiplatelet use^b^	43 (40.9%)	118 (35.8%)	0.38
			
On admission status			
**NIH-Stroke Scale**^c^	**22 (15-32)**	**14 (5-24)**	**<0.01**
**Glasgow Coma Scale**^c^	**7 (3-13)**	**13 (7-15)**	**<0.01**
**ICH-Score**^c^	**3 (2-4)**	**1 (0-3)**	**<0.01**
MAP^a ^(mmHg)	113 (± 27.6)	116 (± 26.7)	0.51
			
Neuroradiological data			
Location; basalganglia^b^	54 (51.4%)	158 (47.9%)	0.64
Location; lobar^b^	44 (41.9%)	131 (39.9%)	0.69
Location; brainstem^b^	5 (4.8%)	21 (6.4%)	0.55
Location; cerebellar^b^	2 (1.9%)	20 (6.1%)	0.12
**ICH volume (cm^3^)**	**48.6 (12.1-82.2)**	**15.0 (4.8-40.6)**	**<0.01**
**IVH**^b^	**74 (70.5%)**	**176 (53.3%)**	**<0.01^**
**Graeb Score**^c^	**4 (0-8)**	**1 (0-4)**	**<0.01**
Hematoma growth^b^	16 (15.2%)	38 (11.5%)	0.31
**Midline shift**^c ^**(mm)**	**6 (2-10)**	**0 (0-5)**	**<0.01**
			
Laboratory values on admission			
**Hemoglobin**^a ^**(mmol/L)**	**6.93 (± 0.81)**	**8.97 (± 0.87)**	**<0.01**
**Hematocrit**^a^	**0.33 (± 0.04)**	**0.41 (± 0.04)**	**<0.01**
Leucocytes^c ^(10^9/L)	8.8 (6.3-12.3)	9.3 (7.2-11.7)	0.34
Thrombocytes^c ^(10^9/L)	235 (153-299)	228 (193-284)	0.44
International Normalized Ratio^a^	1.05 (± 0.18)	1.01 (± 0.06)	0.07
Partial thromboplastin time^a ^(s)	28.4 (± 4.3)	28.7 (± 3.7)	0.62
Creatinine^c ^(μmol/L)	84.9 (64.5-102.6)	81.3 (69.0-97.3)	0.60
C-reactive protein^c ^(nmol/L)	47.6 (9.5-127.6)	29.5 (10.5-76.2)	0.18
Glucose^c ^(mmol/L)	7.49 (6.52-10.07)	7.38 (6.05-9.05)	0.11
			
In-hospital measures			
**Mechanical ventilation**^b^	**55 (52.4%)**	**132 (40.0%)**	**0.03**^d^
Duration of ventilation^c ^(days)	12 (2-22)	12 (3-23)	0.33
**Pneumonia**^b^	**70 (66.7%)**	**163 (49.4%)**	**<0.01**^d^
Sepsis^b^	16 (15.2%)	33 (10.0%)	0.14
Urinary tract infection^b^	11 (10.5%)	35 (10.6%)	0.99
Extraventricular drainage^b^	27 (25.7%)	90 (27.3%)	0.75
Length of stay^c ^(days)	10 (3-20)	11 (6-18)	0.11
**In hospital mortality**^b^	**42 (40.0%)**	**60 (18.2%)**	**<0.01**

^a^Mean (± SD).

^b^*n *(%).

^c^Median (IQR; 25th-75th percentile).

^d^Not sig. after Bonferroni correction.

### Outcome and shift from short- to long-term outcome is worse in anemic patients

**Figure 1:**
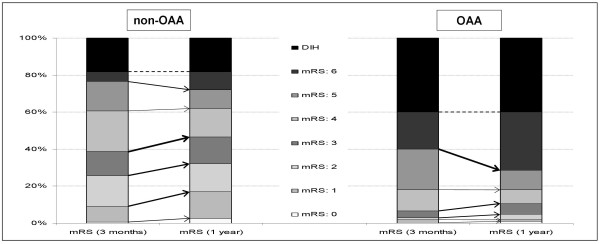
**Dichotomized analysis of the mRS distribution at 90 days and 1 year for non-OAA and OAA patients**. The dashed line indicates, within the mRS = 6 group, the proportion of patients who died in the hospital (DIH). The thickness of arrows indicates the proportional shift from short- to long-term outcome within the mRS groups.

Figure 1 shows the mRS distribution at 90 days and the long-term outcome-shift (at 1 year) among the analyzed patients. In comparison both short- and long-term outcome were significantly poorer in anemic patients (mRS: 4-6 (90 days): OAA = 93.3% *versus *non-OAA = 61.2%, *P *< 0.01; mRS: 4-6 (1 year): OAA = 89.5% *versus *non-OAA = 53.3%, *P *< 0.01) For both groups the proportion of patients showing a favorable long-term outcome increased from 3 months to 1 year. However, the probability of a favorable long-term outcome shift was significant only in non-anemic patients (chance for shift to favorable outcome in non-OAA: OR: 1.5; CI (1.05-2.07); *P *= 0.02 *versus *OR: 1.7; CI (0.61-4.69); *P *= 0.31 in OAA). On the other hand, for anemic patients the probability of a 1-year mortality was higher (OR: 2.4; CI (1.12-5.1); *P *= 0.02) and translated to a more than seven-fold increased risk for an unfavorable long-term outcome (OR: 7.5; CI (3.87-14.48); *P *< 0.01).

### Association of hematoma volume with outcome is less accurate and less relevant in anemia

**Figure 2:**
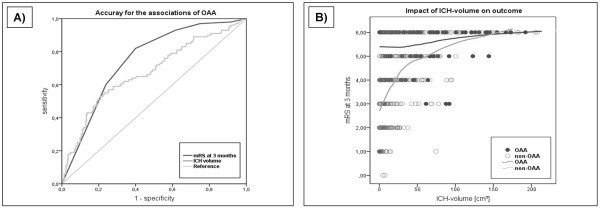
**(A) Receiver operating characteristics curve for the association of OAA with ICH volume and outcome**. (B) Graphical regression analysis; LOWESS graph (a = 0.25-0.5, tension = 66), evaluating the influence of ICH volume on outcome for OAA- and non-OAA patients. Note, that in non-OAA patients hematoma volume is of greater relevance to outcome (especially for volumes < 30 cm^3^) than in OAA-patients.

The reported difference in overall outcome between anemic and non-anemic patients may be theoretically attributed to the different baseline hemorrhage volumes, and corresponding GCS and NIHSS scores, respectively. Therefore, a graphical analysis was performed to analyze the accuracy of the associations for anemia with larger hematoma volumes and functional outcome. Figure 2A shows the ROC curve for the associations of anemia with ICH volume and functional outcome. Anemia revealed a positive but poorer association with larger hemorrhage volumes (AUC = 0.67), whereas the association with functional outcome was positive and more accurate for all spontaneous ICH patients in our study (AUC = 0.75; CI (0.70-0.80); *P *< 0.01).

To investigate the influence of ICH volume on functional outcome for patients with and without anemia, a graphical regression analysis is shown in Figure 2B. The inclination of the curves demonstrates the influence of hematoma volume on outcome, being the stronger the steeper. The OAA curve showed a relatively flat constant slope, regardless of increasing volumes, demonstrating marginal effects of ICH volume on functional outcome. The inclination of the non-OAA curve was considerably steeper, especially for smaller ICH volumes up to approximately 30 cm^3 ^reflecting a stronger influence of hematoma volume on outcome in these 'minor-volume' non-anemic patients. Thereafter, the influence of volume on outcome also decreased in non-anemic patients, revealing a certain volume 'cutoff' after which outcome was likely to be poor in any case.

### Worse outcome in minor-volume ICH patients with anemia despite similar characteristics

In light of the previous investigation proposing anemia-related outcome effects beyond its association with hematoma volume [7], a sub-analysis of minor-volume ICH patients was performed to minimize volume-related effects on outcome. Of the 267 patients with an ICH volume <30 cm^3 ^anemia was present in 42 patients (15.7%). All parameters (as analyzed in Table 1) were tested and the relevant ones shown in Table 2; in particular, the parameters age, hemorrhage volume, and GCS were not statistically different among the compared groups. However, anemic patients appeared to have a tendency towards an increased rate of hemorrhage growth (*P *= 0.07).

**Table 2:** Demographic-, baseline,-, neuroradiological-, laboratory, treatment characteristics for minor-volume ICH patients with OAA *versus *non-OAA.

Minor-volume ICH (*n *= 267)	OAA (*n *= 42)	Ø OAA (*n *= 225)	*P *value
Age^a ^(years)	71.8 (± 11.4)	69.1 (± 12.0)	0.06
On admission status			
			
Glasgow Coma Scale^b^	12 (3-15)	13 (3-15)	0.11
**ICH-Score**^b^	**2 (1-3)**	**1 (0-2)**	**0.01**^c^
MAP^b ^(mmHg)	116 (105-131)	117 (100-133)	0.69
			
Neuroradiological data			
ICH volume^b ^(cm^3^)	8.8 (3.5-14.9)	7.4 (2.9-15.6)	0.64
IVH^d^	23 (54.8%)	105 (46.7%)	0.33
Graeb Score^b^	1 (0-4)	0 (0-3)	0.30
Hematoma growth^d^	9 (21.4%)	25 (11.1%)	0.07
Midline shift^b ^(mm)	0 (0-3)	0 (0-2)	0.10
Laboratory values on admission			
			
International Normalized Ratio^a^	1.05 (± 0.09)	1.04 (± 0.11)	0.49
Partial thromboplastin time^a ^(s)	29.8 (± 3.8)	28.9 (± 3.6)	0.28
			
In-hospital measures			
Mechanical ventilation^d^	19 (45.2%)	77 (34.2%)	0.17
Pneumonia^d^	20 (44.4%)	88 (39.1%)	0.30
**In hospital mortality**^d^	**10 (23.8%)**	**18 (8.0%)**	**0.05**^c^

^a^Mean (± SD).

^b^Median (IQR; 25th-75th percentile).

^c^Not sig. after Bonferroni correction.

^d^*n *(%).

**Figure 3:**
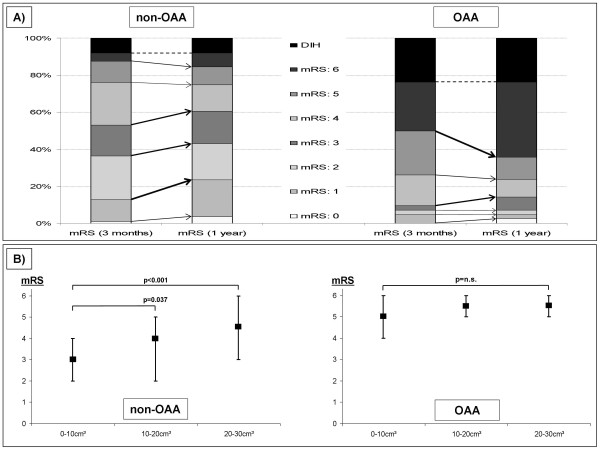
**(A) Dichotomized analysis of the mRS distribution at 90 days and 1 year for non-OAA and OAA patients with minor-volume ICH**. The dashed line indicates, within the mRS = 6 group, the proportion of patients who already died in the hospital (DIH). The thickness of arrows indicates the proportional shift from short- to long-term outcome within the mRS groups **(B) Association of hematoma volume and outcome in non-OAA patients and OAA patients**. Note, that there is a clear association of hematoma volume and outcome only in non-OAA patients.

Despite similar baseline parameters both short- and long-term outcome were also significantly worse in patients with anemia as compared to those without (Figure 3A, mRS: 4-6 (90 days): OAA = 90.5% *versus *non-OAA = 47.1%, *P *< 0.01; mRS: (1 year): OAA = 85.7% *versus *non-OAA = 39.6%, *P *< 0.01). Again for both groups the proportion of patients showing a favorable long-term outcome increased from 3 months to 1 year. Though, statistically not significant, the results were showing the same trends as for the overall cohort: (1) improvement in non-anemic; and (2) increased mortality in anemic patients (*P *= 0.08, *P *= 0.08). The probability of an unfavorable long-term outcome was nine-fold increased for anemic patients (chance of mRS 4-6 OR: 9.2; CI (3.71-22.66); *P *< 0.01).

Figure 3B compares the effects of hematoma volume - stratified volume intervals (10 cm^3^) - on long-term functional outcome. There was a significant association of increasing hemorrhage volume and poor outcome in non-anemic patients, whereas anemic patients did not show this association in minor-volume ICH.

### Anemia is an independent predictor of unfavorable long-term outcome in ICH

In multivariate regression modelling the parameter anemia showed the strongest significant relation to long-term outcome, along with established parameters such as age, hemorrhage volume, GCS, IVH, and presence of hemorrhage growth (Table 3A; OR: 3.0; CI (1.31-7.06); *P *< 0.01). Focussing on patients with minor-volume ICH, multivariate regression analysis revealed an even stronger significant association of anemia with poor outcome (Table 3B; OR: 5.6; CI (1.84-16.85); *P *< 0.01). Interestingly, in minor-volume ICH patients parameters such as hemorrhage volume and ventricular involvement did not hold significance when adjusted for anemia.

**Table 3:** Multivariate regression models for predictors of unfavorable (mRS = 4-6) functional outcome at 1 year.

Multivariate - parameters	Unfavorable outcome Odds ratio (95% CI)	*P *value (*P *< 0.05)
**A) Overall ICH patients**		
**Age **	**1.102 (1.070-1.136)**	**<0.0001**
**Volume **	**1.022 (1.007-1.038)**	**0.0050**
**GCS**	**0.858 (0.788-0.934)**	**0.0004**
**IVH (Graeb Score)**	**1.140 (1.031-1.261)**	**0.0108**
**Hematoma growth**	**2.903 (1.188-7.093)**	**0.0194**
Diagnosis of hypercholesterolemia	0.809 (0.435-1.505)	0.5030
Ventilation	1.030 (0.996-1.064)	0.0807
**On admission anemia**	**3.045 (1.314-7.058)**	**0.0094**

**B) Minor-volume ICH patients**		
**Age **	**1.102 (1.062-1.144)**	**<0.0001**
Volume	1.032 (0.990-1.076)	0.1345
**GCS**	**0.755 (0.668-0.854)**	**<0.0001**
IVH (Graeb Score)	1.039 (0.920-1.174)	0.5388
**Hematoma growth**	**4.646 (1.561-13.831)**	**0.0058**
Diagnosis of hypercholesterolemia	0.711 (0.334-1.513)	0.3761
Ventilation	1.015 (0.970-1.063)	0.5126
**On admission anemia**	**5.573 (1.843-16.853)**	**0.0023**

## Discussion

In the present study we analyzed associations of anemia with hemorrhage volume and its impact on functional outcome in patients with spontaneous ICH. Overall, the presence of anemia is high and affects almost one-quarter of these patients. Anemia was associated with larger baseline hemorrhage volumes and worse outcome as compared to patients without. However, anemia revealed independent effects on functional outcome beyond the - at first glance trivial - association with hematoma volume. Especially, in patients with minor-volume ICH with an increased likelihood of favorable functional recovery, anemia impacted outcome independently and to a larger degree than established parameters. Several aspects deserve attention.

### Prevalence and general aspects

The prevalence of anemia in the community-dwelling population of patients aged >65 years is >10%, increases profoundly with age and is often multifactorial [16]. Nutritional deficiency anemia, anemia of inflammation, and anemia of unknown causes are the most frequent sub-types [16]. Anemia, in general, is known through large population-based studies to contribute to a higher likelihood of an unfavorable outcome and increased mortality [27-29]. Focussing on ICH patients who are severely diseased with a high mortality rate and poor prognosis *per se*, it seems rational that the hemorrhage itself attracts much of the medical attention [30]. However, in light of almost 25% of ICH patients presenting with anemia and given its independent impact on outcome, as reported here, it seems necessary to improve recognition and treatment of anemia on stroke and neurocritical care units.

### Overall impact of anemia on hemorrhage volume and outcome

In line with Kumar and colleagues [7], patients with anemia showed an association with larger ICH volumes. There are several studies that established the influence of anemia and abnormal coagulation, hemostatic alterations, and an increased bleeding tendency [31-33]. Our findings support the necessity to verify this small body of evidence - corroborating the belief of anemia-associated increased ICH volumes - by analyses of larger patient cohorts from RCTs. Moreover, there may be independent effects of anemia on outcome that act beyond ICH volume establishing anemia itself as a condition of general morbidity and increased mortality. Our results for anemic patients convey less functional recovery and a greater proportion of patients passing away during the course after ICH. Therefore, anemia seems to be related to consecutive clinical detoriation in spite of the generally accepted improvements that occur over time [34]. The recognition of anemia has increased across various medical specialties within the emerging concepts of ageing and frailty. Anemia may possibly be more than a contributor to detoriation after ICH and also be relevant to the neurological status upon initial presentation [35-38]. Again, detection and treatment of anemia seems easily doable and its influence on outcome in ICH needs urgently to be determined.

### Anemia identifies patients at risk for poor outcome in minor-volume ICH

The relevance of anemia is even more striking in minor-volume ICH patients. Despite similar baseline clinical and radiological characteristics anemia strongly impacted outcome. Especially, considering the outcome-shift in anemic patients resulted in a higher probability of unfavorable long-term outcome. The only meaningful explanation for the observed outcome difference is the anemic condition itself. Though, anemia was not as prevalent in minor-volume ICH, as compared to the entire cohort, focusing on hemoglobin levels upon admission may help identifying high-risk patients with co-morbidity and increased risk for hemorrhage growth. Latter as an important predictor of outcome in ICH [21] which may theoretically be influenced specifically in anemic patients by hemostatic drugs [30]. Other detrimental anemia-based mechanisms refer to neuronal tissue hypoxia, metabolic distress, and cell energy dysfunction that possibly lead to pronounced secondary cerebral injury by a reduced oxygen carrying capacity. [4,39-41].

### Treatment of anemia during acute phase?

In neurocritical care acute treatment strategies using RBC transfusions and its appropriate thresholds remain controversially debated resulting in widespread practice variations [2,4,8,42]. Recently, Sheth and colleagues presented a large retrospective study describing a decreased mortality for anemic ICH patients who received RBC transfusions during hospital stay [8], although exact transfusion regimes have not been investigated. Therefore, in regard of the existing evidence of conventional transfusion strategies for general ICU patients (TRICC study), a more liberal transfusion regime has to be very cautiously evaluated in each patient and also balanced against ethical concerns because of blood product shortage [2,5]. Moreover, disease entities are different in neurocritical care requiring specific approaches which may not be generalized [1,2,4]. For instance, in SAH the value of anemia and its possible compensation by RBC transfusions has not been entirely clarified [2,43]. It is indispensable to investigate the pros and cons of administering RBC transfusions with its specific thresholds in neurocritical care, especially in ICH. Only a well-designed prospective interventional study may resolve the question if compensating lowered hemoglobin levels affects mortality and functional outcome. In any case it should be focused on minor-volume ICH patients with a greater chance of recognizing treatment effects.

Our study has a number of limitations: (1) the retrospective, single-center design; (2) the mailed questionnaires may have been answered wrongly with respect to the time-point of follow-up and validity of mRS estimation [44]; (3) hematologic investigations for the different etiologies of anemia have not been applied; (4) the results may have been biased by the 29 patients lost to follow-up; (5) although anemia was an independent outcome-predictor, residual confounding by parameters not investigated as well as interactions between poorer neurological admission status and outcome may not be fully excluded; (6) moreover, ICH itself may hypothetically cause anemia which however seems unlikely according to available data [45]; (7) finally, our study descriptively analyzed relations of anemia and outcome. Given the study design we were not able to contribute to a better understanding of underlying causes, notably a biologically plausible mechanism of how outcome is influenced by an anemic condition.

## Conclusions

Within this observational cohort study, anemia is independently associated with unfavorable functional outcome in ICH beyond its relationship with larger hemorrhage volumes, the latter being basically less relevant in anemic patients. This also holds true for rather therapeutically relevant patients with minor-volume ICH and good chances for recovery. Prospective trials with precise hematological evaluations and interventions are warranted to clarify potential treatment options in patients with anemia and ICH.

## Key messages

• Anemia is positively but poorly associated with larger hemorrhage volumes.

• Anemia is a previously unrecognized independent predictor of unfavorable functional outcome with an odds ratio of 3.

• Negative long-term outcome-shift in patients with anemia; → seven times more likely to have unfavourable long-term outcome.

• Minor-volume ICH anemia exerted even stronger independent effects on outcome with an odds ratio of 5.6.

• Prospective trials with precise hematologic assessment and interventions are warranted in ICH patients.

## Abbreviations

AUC: area under the curve; CI: confidence interval; EVD: external ventricular drainage; GCS: Glasgow Coma Scale; GS: Graeb score summation; Hb: hemoglobin; ICH: intracerebral hemorrhage; INR: international normalized ratio; IQR: interquartile range; IVH: intraventricular hemorrhage; LOWESS: locally weighted scatterplot smoothing; mRS: modified Rankin Scale; NIHSS: National Institute of Health Stroke Scale; OAA: on admission anemia; OR: odds ratio; RBC: red blood cells; RCT: randomized controlled trials; ROC: receiver operating characteristics curve; SAH: subarachnoid hemorrhage; TBI: traumatic brain injury.

## Competing interests

The authors declare that they have no competing interests. The study was not funded.

## Authors' contributions

JBK, MK, and HBH designed the study and wrote the manuscript. JBK and SG obtained clinical data by reviewing institutional databases and the patients' medical charts. HL and SK obtained all neuroradiological data. JBK, SG, and MK obtained the pre-admission and functional outcome data of all patients. PDS performed the statistical analyses and critically revised the manuscript. All authors have read the manuscript, agreed with the contents, and approved the final version of the manuscript.
